# Population‐level prokaryotic community structures associated with ferromanganese nodules in the Clarion‐Clipperton Zone (Pacific Ocean) revealed by 16S rRNA gene amplicon sequencing

**DOI:** 10.1111/1758-2229.13224

**Published:** 2023-12-26

**Authors:** Kento Tominaga, Hiroaki Takebe, Chisato Murakami, Akira Tsune, Takahiko Okamura, Takuji Ikegami, Yosuke Onishi, Ryoma Kamikawa, Takashi Yoshida

**Affiliations:** ^1^ Graduate School of Agriculture Kyoto University Kyoto Japan; ^2^ Deep Ocean Resources Development Co., Ltd. Tokyo Japan; ^3^ KANSO TECHNOS Co., Ltd. Osaka Japan

## Abstract

Although deep‐sea ferromanganese nodules are a potential resource for exploitation, their formation mechanisms remain unclear. Several nodule‐associated prokaryotic species have been identified by amplicon sequencing of 16S rRNA genes and are assumed to contribute to nodule formation. However, the recent development of amplicon sequence variant (ASV)‐level monitoring revealed that closely related prokaryotic populations within an operational taxonomic unit often exhibit distinct ecological properties. Thus, conventional species‐level monitoring might have overlooked nodule‐specific populations when distinct populations of the same species were present in surrounding environments. Herein, we examined the prokaryotic community diversity of nodules and surrounding environments at the Clarion‐Clipperton Zone in Japanese licensed areas by 16S rRNA gene amplicon sequencing with ASV‐level resolution for three cruises from 2017 to 2019. Prokaryotic community composition and diversity were distinct by habitat type: nodule, nodule‐surface mud, sediment, bottom water and water column. Most ASVs (~80%) were habitat‐specific. We identified 178 nodule‐associated ASVs and 41 ASVs associated with nodule‐surface mud via linear discriminant effect size analysis. Moreover, several ASVs, such as members of SAR324 and *Woeseia*, were highly specific to nodules. These nodule‐specific ASVs are promising targets for future investigation of the nodule formation process.

## Graphical abstract

We examined population‐level prokaryotic community diversity in nodules found in the Clarion‐Clipperton Zone of the Pacific Ocean by 16S rRNA gene amplicon sequencing with amplicon sequence variant (ASV)‐level resolution extracted from sequence polymorphism. Prokaryotic community composition and diversity was distinct by habitat type, nodules, nodule‐surface mud, sediment, bottom water, and water column. We identified and characterized several ASVs such as ASVs closely related to SAR324 and Woeseia, highly specific to nodules and nodule‐surface mud.

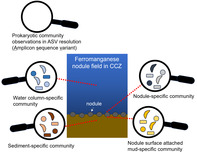

## INTRODUCTION

Ferromanganese (Fe‐Mn) nodules, composed mostly of iron and manganese oxides (Fe‐Mn oxides), are spherical marine sedimentary mineral deposits (Glasby, 2006; Hein & Koschinsky, 2013). Owing to the high concentrations of economically valuable elements (such as Ti, Co and Ni), these Fe‐Mn nodules (or Fe‐Mn crusts, which are another form of Fe‐Mn oxides found on the seafloor with different shapes, formation depths and chemical compositions) have attracted economic interest as potential mineral resources (Miller et al., 2018). However, the process of Fe‐Mn nodule/crust formation remains unclear because of the slow growth of nodule formation (e.g. 1 cm/millions years) (Koschinsky & Halbach, 1995; Usui et al., 2017). Since Fe–Mn nodules and crust harbour diverse and abundant prokaryotic microorganisms (estimated to be 10^6^–10^8^ cells/g) (Kato et al., 2018; Lindh et al., 2017; Nitahara et al., 2017; Shiraishi et al., 2016; Shulse et al., 2017), prokaryotic activity has been suggested to be involved in the elemental transition between seawater and Fe‐Mn substrates via Mn oxidation and Mn precipitation (Blöthe et al., 2015; Kato et al., 2019; Wang & Müller, 2009; Wu et al., 2013). Therefore, identifying prokaryotic populations associated with nodules and elucidating their physiological and ecological characteristics is necessary to understand the Fe‐Mn nodule formation process.

To date, prokaryotic community assessments of Fe‐Mn nodule/crust fields have led to the identification of prokaryotic taxa that might be associated with these mineral deposits (Blöthe et al., 2015; Cho et al., 2018; Kato et al., 2018; Liao et al., 2011; Lindh et al., 2017; Nitahara et al., 2011, 2017; Shulse et al., 2017; Tully & Heidelberg, 2013; Wang et al., 2010; Wang & Müller, 2009; Wu et al., 2013). They were determined to be important in energy and geochemical cycling at nodule/crust, and thus, proposed as taxa that contribute to the formation and maintenance of Fe–Mn substrates. However, in most of the previous studies, they were described in species‐level operational taxonomic units (OTUs) based on 16S rRNA gene sequence identity. Recent methodological developments have enabled the resolution of amplicon sequence variants (ASVs), which discriminates sub‐populations within a species‐level OTU based on polymorphism, via statistical analysis of high‐throughput sequence data (Amir et al., 2017; Callahan et al., 2016; Edgar, 2016; Eren et al., 2013, 2015; Tikhonov et al., 2015). Such ASV‐level analyses revealed that OTUs often include multiple populations having different ecologies (Callahan et al., 2017; Chafee et al., 2018; Needham et al., 2017). A previous study in nodule field of CCZ pointed out the importance of ASV‐level resolution assessment of community diversity (Wear et al., 2021). However, the study was relatively low‐sequence coverage (rarified to 6900 reads per sample), and there were only a few other ASV resolution studies in other Fe‐Mn crusts/nodules and surrounding environments had been performed to date (Bergo et al., 2021). Thus, important nodule‐specific prokaryotic populations might have been overlooked if OTUs of nodules/crusts were shared with surrounding environments.

In this study, we analysed ASV‐resolution prokaryotic diversity at nodule samples, surrounding sediments and water column by 16S rRNA gene amplicon sequencing at Clarion‐Clipperton Zone (CCZ; Pacific Ocean) in Japanese licensed mining area for 3 years. We also assessed the long‐term environmental impacts of the sediment resuspension and redeposition resulting from deep‐sea mining on benthic prokaryotic communities.

## EXPERIMENTAL PROCEDURES

### Sampling

Seawater, sediment, nodules and surface mud adhered to nodule samples were collected during three different sampling cruises at CCZ in Japanese licensed mining area by Deep Ocean Resources Development Co., Ltd., three times: 20 October 2017 to 29 October 2017 (1st samplings) and 21 September 2018 to 1 October 2018 (2nd samplings), and 15 August 2019 to 20 August 2019 (3rd samplings). St. E, St. C and St. W. are the stations that were placed to monitor the change of the marine and sediment environment in the contract area (Nishi et al., 2018), and PR01 is located within called Preservation Reference Zone (Jones et al., 2020; Lodge et al., 2014) in the contract area. In 1994, at the JET site, the “Japan Deep‐Sea Impact Experiment (JET)” was performed to evaluate the effects of sediment resuspension and redeposition resulting from deep‐sea mining (Fukushima, 1995; Fukushima et al., 2022; Fukushima & Tsune, 2019). In the experiment, the same benthic disturber in the Benthic Impact Experiment (BIE) was used towed over 19 transects in the experimental area, discharging around 350 tons (dry weight) of sediment, which reached thicknesses of up to 19.5 mm on the seafloor (Fukushima, 1995). Note that the objective of this study was not to assess the impact of JET experiment but to compare ASVs between the nodules and the surrounding environment. Furthermore, since no clear effect of the disturbance experiment was observed, it was not discussed separately from the other samples in most of the analyses (see Results). The following three areas of the JET site (Fukushima et al., 2022) were selected as sampling stations for this study: (1) heavy deposition area (J6‐08), where the disturbance test was conducted in 1994; (2) reference area (J6‐14), which was the control area where no disturbance effect was considered; and (3) medium or light deposition area (J6‐10), which is located between the heavy deposition area and the reference area. Maps of the sampling stations are shown in Supporting Figure S1, and details of each sample are shown in Supporting Table S1.

The nodules and sediments were sampled using a multiple corer sampler and box corer sampler. For sediment sampling, during the 1st sampling, sediment samples were collected from the 0–0.5 cm surface layer with a sterile spatula into DNA‐free 15‐mL volumetric centrifuge tubes. During the 2nd sampling, sediment samples were collected from the 0–0.5, 0.5–1.0, 1.0–2.0, 2.0–3.0, 3.0–4.0, 4.0–5.0, 5.0–6.0, 6.0–8.0 and 8.0–10 cm layers. In the 3rd sampling, sediment samples were collected from the 0–0.5, 0.5–1.0, 1.0–2.0, 2.0–3.0, 3.0–4.0 and 4.0–5.0 cm layers (with triplicates). For nodule sampling, the nodules were scraped with a sterile stainless‐steel grater and the scraps were collected. The side of the nodule facing the seawater was designated as the “front side” and the side of the nodule buried in the sediment was designated as the “bottom side.” For sampling the mud adhering to the surface of the nodules (hereafter referred to as nodule‐surface mud), the nodules were washed off with filter‐sterilized seawater, and the water containing the muds was collected. The sediment, nodule and nodule‐surface mud samples were stored in sterile plastic bags (UniPack) and frozen at −80°C until DNA extraction. For seawater sampling, seawater was collected at the surface using a bucket and at depths of 50, 100, 150, 200, 300, 500, 750, 1000, 2000, 3000, 4000 and 5000 m using Niskin water samplers. For the analysis of bottom water communities just above the seafloor, the seawater remaining in the box corer or seawater sampled by a 5 L‐Niskin water sampler attached to the frame of the multiple corer sampler was collected. The obtained seawater (2–8 L) was filtered through a 0.2‐μm pore size Sterivex filter (Merck) and frozen at −80°C until DNA extraction. Altogether, for prokaryotic community analysis, 142 samples (72 of sediment, 26 of water column, 26 of nodules, 10 of bottom water and 8 of samples) were obtained.

### 16S rRNA gene amplicon sequencing

Nodule, nodule‐surface mud and sediment (0.5 g) samples were thawed, and DNA was extracted using the ISOIL for Beads Beating kit (Nippon Gene, Tokyo, Japan) according to the manufacturer's protocol. For the water samples, DNA was extracted from the stored filtration units using a previously described protocol (Takebe et al., 2020). The V3‐V4 hypervariable region of the 16S rRNA gene was amplified by PCR using primers 341F (5′‐CCTACGGGRSGCAGCAG‐3′) and 805R (5′‐GACTACCAGGGTATCTAAT‐3′), with overhang adapters (forward: TCGTCGGCAGCGTCAGATGTGTATAAGAGACAG, reverse: GTCTCGTGGGCTCGGAGATGTGTATAAGAGACAG) (Takahashi et al., 2014). Libraries were prepared according to the 16S Metagenomic Sequencing Library Preparation guide (#15044223 Rev. B; Illumina, San Diego, CA, USA). Paired‐end (2 × 300 bp) sequencing was conducted using an MiSeq platform (Illumina).

### Sequence analysis

After trimming adapters from raw reads with q2‐cutadapt, we used the DADA2 plugin (Callahan et al., 2016) of QIIME2 version 2020.11 (Bolyen et al., 2019) to perform quality filtering, denoising and pair‐end merging, and construct a feature table of ASVs. Multiple sequence alignment of the sequences was performed using MAFFT (Katoh et al., 2002). The aligned dataset was used to reconstruct an approximately Maximum‐Likelihood phylogenetic tree with FastTree, using the default settings of the QIIME phylogeny plugin (Price et al., 2010). The taxonomy of the ASVs was assigned using Naive Bayes classifiers trained on 99% OTUs of full‐length sequences in SILVA 138 (Quast et al., 2013) by q2‐feature‐classifier plugin (Bokulich et al., 2018).

### Phylogenetic placement

For reference trees, multiple sequence alignment of the 16S rRNA gene sequences collected in previous studies (Boeuf et al., 2021; Hoffmann et al., 2020) was performed using MAFFT with the—maxiterate 1000—localpair option (Katoh et al., 2002). The aligned dataset was used to reconstruct an approximately Maximum‐Likelihood phylogenetic tree with FastTree under the GTR model (Price et al., 2010). Phylogenetic placement analysis of abundant ASVs was performed using pplacer (Matsen et al., 2010), and the best hit clade of each ASV was extracted using Gappa (Czech et al., 2020).

### Statistical analysis

All sequence data were rarefied to 54,000 sequences per sample, which was the lowest sample depth, for diversity analysis. For the analysis of Shannon diversity, estimated richness (Chao1) and evenness (Pielou's J) were estimated using “vegan” package in R (Oksanen et al., 2020). Beta diversity was assessed by principal coordinate analysis (PCoA) using the “vegan” package and hierarchical clustering using the “hclust” package in R based on Bray–Curtis dissimilarity. Kruskal‐Wallis rank sum test for the differences in alpha diversity among more than two different sampling stations and habitat types, their pairwise comparisons using the Wilcoxon rank sum test with *p*‐values adjusted by the Bonferroni method, and PERMANOVA for the differences in beta diversity among more than two sampling stations or types were performed using the R package “vegan” (Dixon, 2003). When beta diversity of two sampling stations or habitant types were compared, pairwise PERMANOVA test with *p*‐values adjusted by the Holm method was performed using “pairwiseAdonis” package (https://github.com/pmartinezarbizu/pairwiseAdonis) (Arbizu, 2021). Significant differences between different habitats (nodule, nodule‐surface muds, sediment, bottom water and water column) in terms of relative ASV abundance were determined by linear discriminant analysis (LDA) effect size (LefSe, Segata et al., 2011) with default cut‐off parameters (LDA >2.0, *p* < 0.05). The analysed sequences and statistical data were visualized in R using “ggplots2” (Wickham, 2009).

## RESULTS

### Prokaryotic alpha diversity comparison by habitat type and sampling stations

**FIGURE 1:**
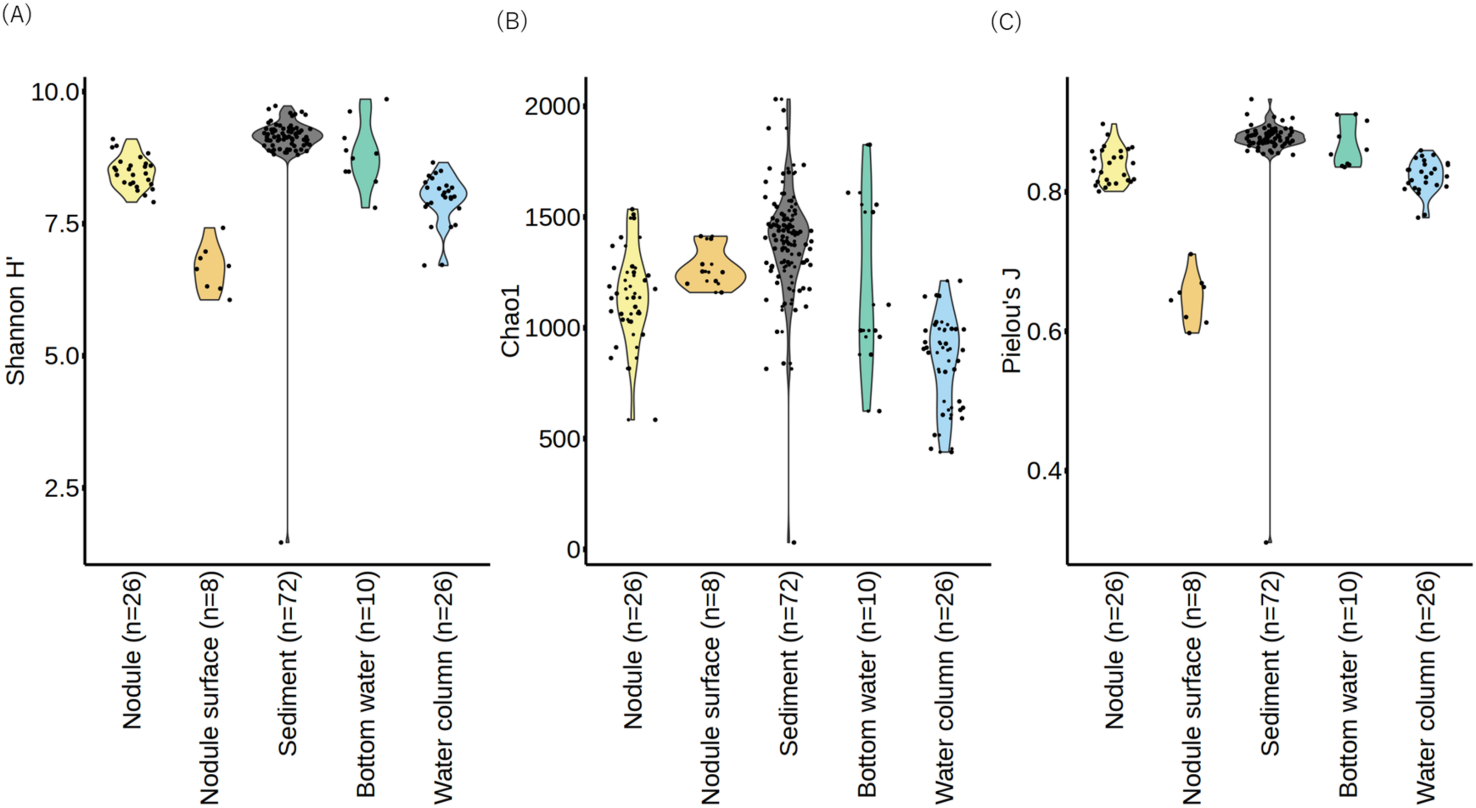
Alpha diversity indices in different habitat type (i.e., nodule, nodule‐surface mud, sediment layers, bottom water, and water column). (A) Shannon H′ (richness and evenness); (B) Chao1 (richness); (C) Pielou's J (evenness).

A total of 25,960,869 sequences were retrieved from 142 samples, and 40,327 ASVs were obtained from quality‐filtered reads (Supporting Table S2). First, we compared the alpha diversity of samples originating from different habitat types (i.e., nodule, nodule surface‐ mud, sediment, bottom water and water column). Alpha diversity metrics (Shannon H′; Figure 1A) were generally higher in sediments than nodules or water columns except for one sample (replicate 2 of 0–0.5 cm layer from the sediment surface at J6‐08). Because Shannon H′ is a composite of richness and evenness, we analysed each of them separately. Although nodule‐surface muds had the second highest estimated species richness (average Chao1 = 1272, Figure 1B), the overall alpha‐diversity was the lowest (average Shannon H′ = 6.65, Figure 1A) because of the lowest species evenness (Prelou's J = 0.65, Figure 1C). Alpha diversity of nodule samples was significantly higher than that of nodule‐surface muds or water columns and significantly lower than that of sediments (pairwise Wilcoxon signed rank test, *p* < 0.01, Figure 1, Supporting Table S3). The estimated species richness in the water column was lower in the surface layer than in the deep layer (Supporting Figure S2A–C). In the outlier sample of sediment, only 31 ASVs were observed (cf. average Chao1 of sediment samples = 385), and ASV_11 (classified into *Delftia*) was exclusively dominant (exceeding >80% read counts of the sample). Th outlier sample was only one sample below the detection limit of DNA concentration. This may be a technical error of DNA extraction, but it is also possible that an uncommon sample showed very low biomass and diversity; we analysed both cases by including/excluding this sample for the diversity assessment, if necessary, in the following section.

Next, we compared the alpha diversity among different sampling stations with the same habitat type. Significant differences in alpha diversity between different sampling stations were observed only in the sediment samples (Supporting Figure S3A–C, Supporting Table S3, Kruskal‐Wallis rank sum test, *p* < 0.01). Although the difference was significant regardless of whether including or excluding the outlier sample (replicate 2 of the 0–0.5 cm layer from the sediment surface at J6‐08), it was smaller than the difference between habitat types (Supporting Figure S3, Supporting Table S3). These alpha diversity analyses suggest that prokaryotic communities differ between habitat types but have no large differences among samples with the same habitat types from different stations. No significant difference was observed between the heavy deposition area of the JET experiment (J6‐08) and the reference area (J6‐14).

### Prokaryotic community composition differed by habitat type

**FIGURE 2:**
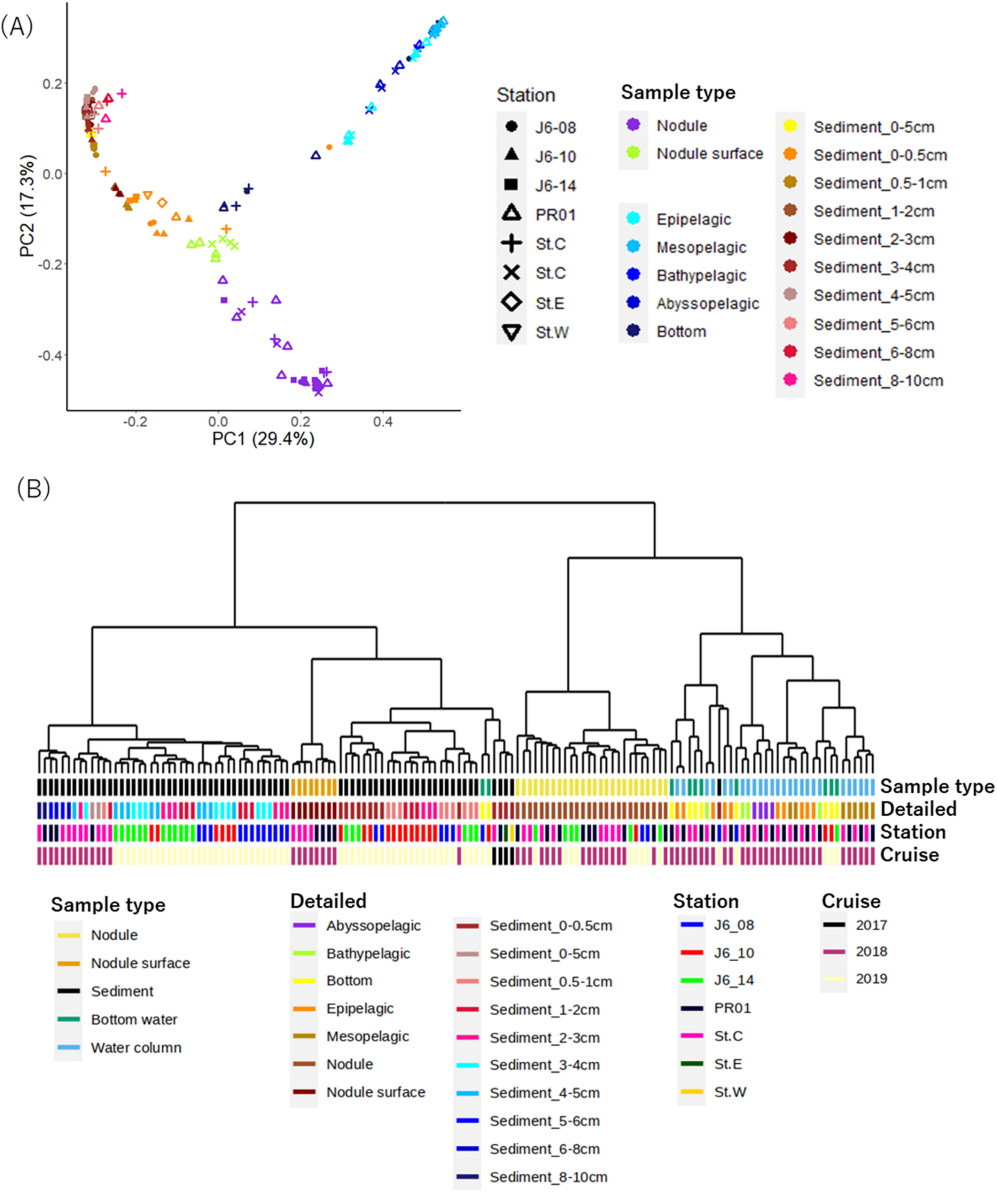
Prokaryotic community beta diversity analysis calculated from Bray‐Curtis distances. (A) Principal coordinate plot of samples based on prokaryotic community dissimilarity (beta diversity) calculated from Bray‐Curtis distances. The colour and shape of points describe the sample type and station, respectively. (B) Hierarchical clustering of samples based on Bray‐Curtis distances. The colour bars under the dendrogram describe the sample type, detailed classification of sample type, sampling stations and sampling year, respectively.

To further characterize the differences in community composition among samples derived from different habitat types, we performed beta diversity analyses between samples based on the Bray‐Curtis distance. Dissimilarity between samples was visualized using PCoA analysis (Figure 2A) and hierarchical clustering based on the bray–Curtis similarity between samples (Figure 2B). In both methods, samples from the same stations were not often clustered, rather most of the samples belonging to the same habitat type were clustered (Figure 2A,B), suggesting that community compositions were more similar among samples belonging to the same habitat type, even from different stations, than among samples belonging to different habitat types from the same stations. We also confirmed that the differences in community composition among the habitat types from the same stations were statistically significant using the PERMANOVA test (*p* < 0.01; Supporting Table S3). Statistically significant differences were detected in almost all pairs of habitat types, except for pairs between nodule‐surface muds and sediment and between the water column and bottom water (pairwise comparison by PERMANOVA adjusted by Holm method, *p* < 0.01, Supporting Table S3). In contrast, the difference between stations was not obvious in either plotting method (Figure 2A,B) and the PERMANOVA exhibited a weaker statistical significance (Supporting Table S3, *p* = 0.022). While this is not a sufficient comparison due to the difference in sampling method in each year, within the same sample type, the samples obtained in the same year are closer together on the dendrogram than samples from the same locations in other years even though they took from different sites (Figure 2B), may suggest that there were temporal changes in the community. As in the case of alpha diversity, samples with disturbance by the JET experiment were not clearly separated from those without disturbance (Figure 2), and their difference was not statistically significant (Supporting Table S3). This suggest influence of the JET disturbance experiment on prokaryotic community composition was not detectable in our observation. Therefore, we did not consider the influence of the disturbances in subsequent analyses.

### Characterization of ASVs

**FIGURE 3:**
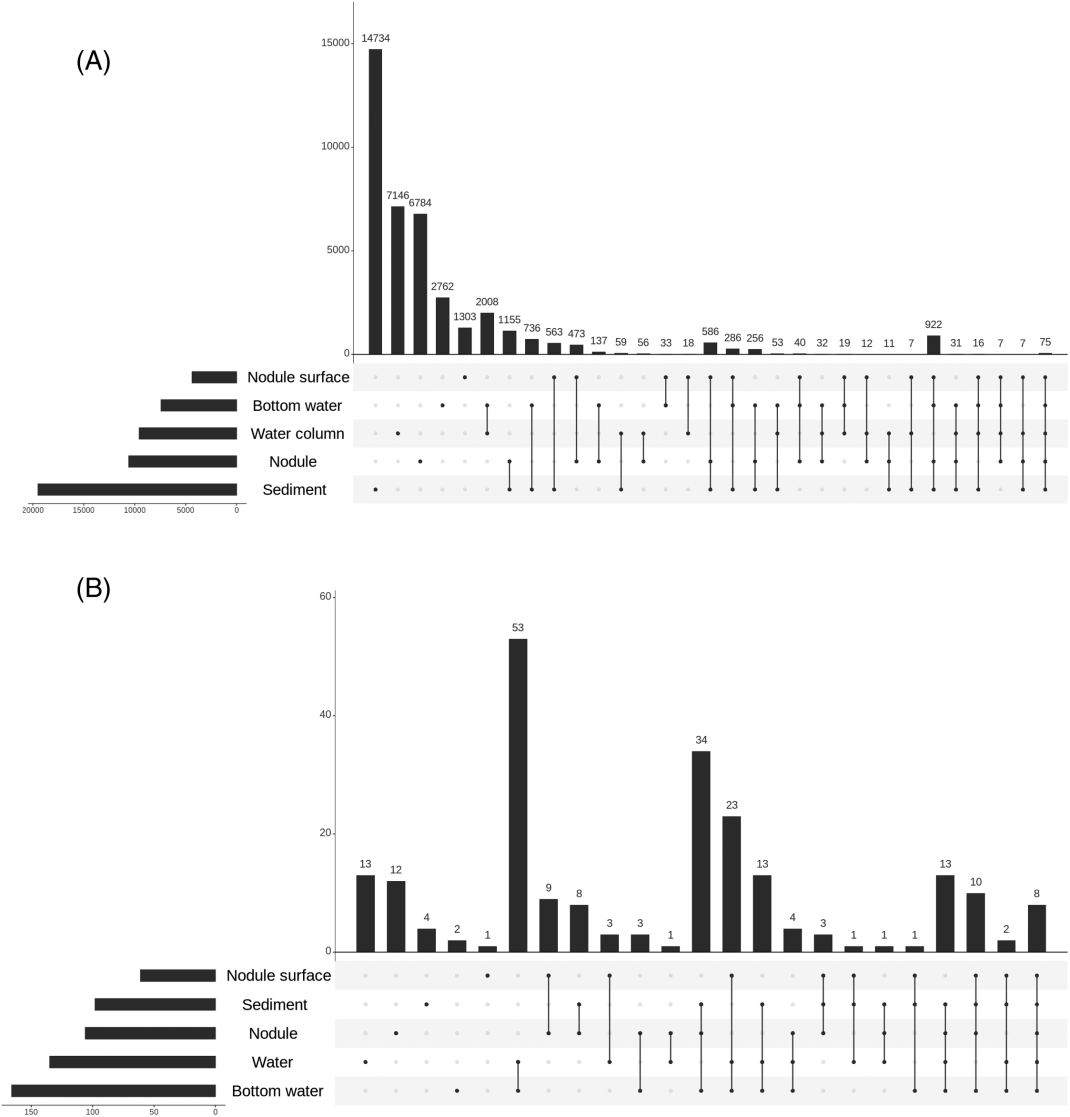
Upset plot showing the distribution of amplicon sequence variants (ASVs) and the number of shared ASVs between habitats. (A) All ASVs; (B) Only abundant ASVs (exceeding >1% relative abundance at least a sample).

To further evaluate differences in community composition among samples derived from different habitat types, we compared the ASV members in each sample type. Consistent with overall significant differences in the beta‐diversity analysis (above), the majority of the observed ASVs were unique (~80%) in either of habitat types, but only 75 ASVs were commonly observed in all the habitat types (Figure 3A). In particular, members of communities in the nodule‐surface mud and nodules were distinct from each other at the ASV level, even though those habitats were physically adhered. Only 473 ASVs were specifically shared between those habitat types (c.f., 1303 and 6784 ASVs were specific to the nodule‐surface mud and nodules, respectively; Figure 3).

We also searched for sampling station‐specific ASVs (Supporting Figure S4). We observed 26,379 ASVs specifically in single sampling stations, although more than 2000 ASVs were shared by all sampling stations (Supporting Figure S4A). Given the smaller number of ASVs shared by all habitat types than that of ASVs shared by all stations, the effect of geographic distance is highly likely smaller on prokaryotic population compositions than that of habitat type, as suggested by the beta diversity analysis performed above. However, when we focused on ASVs exceeding 1% relative abundance in at least one sample (hereafter called abundant ASVs), which comprised 221 ASVs in total, we determined that they were more frequently shared between two or more habitat types or stations than non‐abundant ASVs (Figure 3B, Supporting Figure S4B), presumably because of their ubiquity and abundance in the sampling areas. Alternatively, abundant populations detected as abundant ASVs might easily diffuse from the originally dominated habitats.

To characterize the qualitative differences in diversity among the samples derived from different habitat types, we further compared the prokaryotic taxonomic compositions of the samples. Even with a high taxonomic rank (phylum or class level of Proteobacteria), prokaryotic composition varied among habitat types. Proteobacteria (mostly γ‐proteobacteria and α‐proteobacteria), Bacteroidetes and Planctomycetes were dominant in all habitat types. However, the proportion of Proteobacteria (especially γ‐proteobacteria) was higher in the nodule and nodule‐surface mud samples (Supporting Figure S5). On average, 41.5% and 69.2% of reads in nodule and nodule surface samples, respectively, were γ‐proteobacteria. Particularly, members of Alteromonadales and Oceanospirillales exceeded more than 60% in the nodule‐surface mud samples (Supporting Figure S5). Marinimicrobia (SAR406_clade) was dominant only in the water samples (water column or bottom water). The SAR324 clade was also more abundant in water samples, but it was also one of the abundant phyla in nodule samples. Chloroflexi and Crenarchaeota were more dominant in the sediment samples than in the nodule or nodule surface mud.

### Identification of ASVs associated with nodules and nodule‐surface muds

**FIGURE 4:**
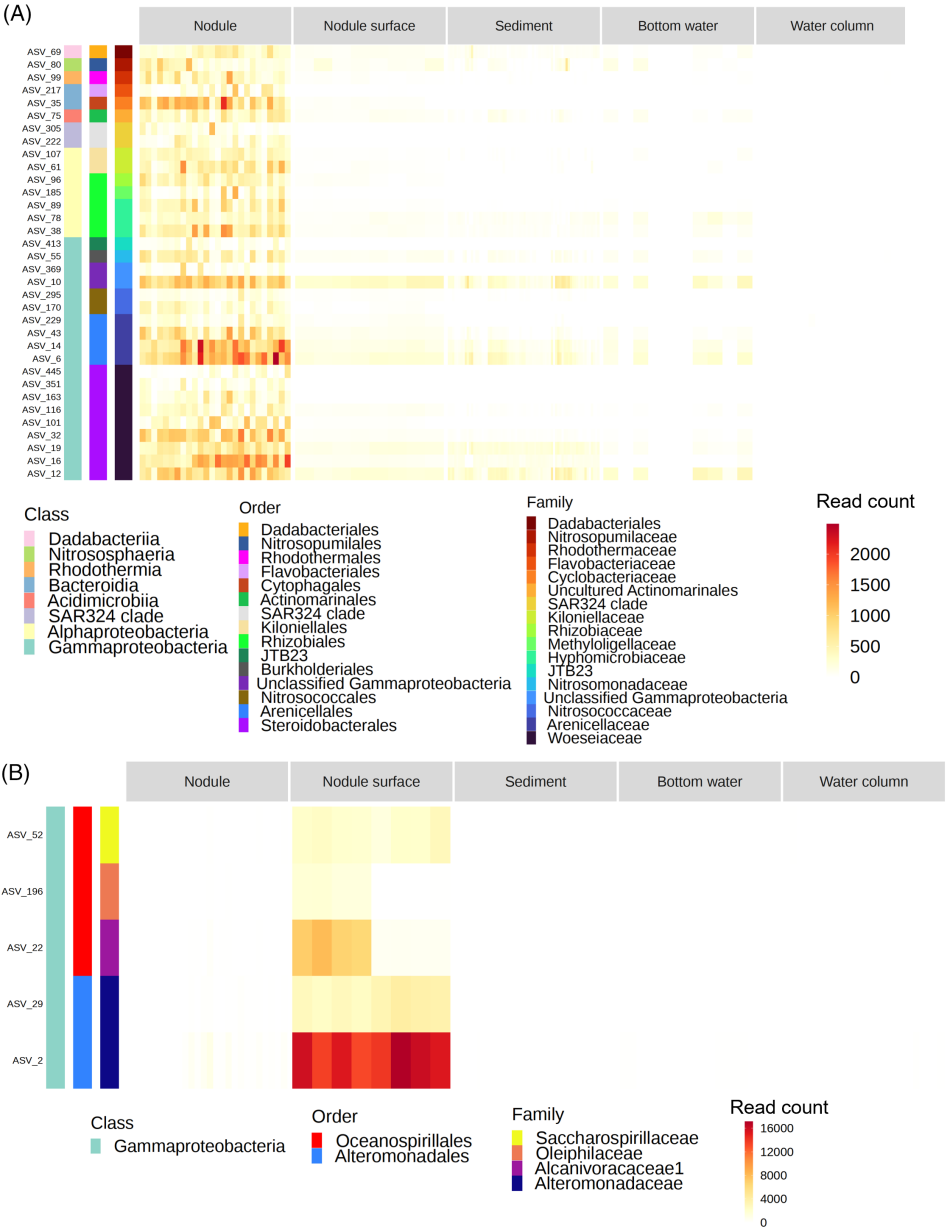
Nodule and nodule surface attached mud associated abundant ASVs detected by linear discriminant effect size (LefSe) and its abundance in each habitat. (A) Nodule associated abundant ASVs; (B) Nodule surface mud associated ASVs. Taxonomy of each ASV was shown in the left colour bars (Class level to Family level).

If microbial activity is important in nodule formation, as suggested in previous studies, some of the members of these taxa that dominate nodules (or nodule surface‐attached muds) seem to be potential contributors to the activity. Thus, to obtain the ASVs associated with specific habitats, especially for nodules and nodule‐surface mud, we attempted linear discriminant effect size analysis to detect them (Figure 4A,B, Supporting Figure S6A,B). In total, 312 ASVs were specifically abundant in single habitats (Supporting Figure S6). Especially, 178 ASVs, including 34 abundant ASVs that exceeded 1% relative abundance in at least one sample, and 41 ASVs, including 5 abundant ASVs, were detected as ASVs associated to nodule (Figure 4A).and nodule‐surface mud, respectively (Figure 4B). Since the abundant ASVs are more plausible candidates than rare ASVs as contributors of geochemical cycling, which might result in nodule formation in the habitats, we further focused on the abundant ASVs in the nodules for the following analyses. All the abundant ASVs associated to the nodules were assigned to either of the following 7 lineages: γ‐proteobacteria (19 ASVs, 9 of which were members of *Woeseia*, formerly called JTB255‐Marine Benthic Group, 4 were members of an uncultured genus of *Arenicellaceae*, and 6 were others), α‐proteobacteria (7 ASVs, all of which were members of either Kiloniellales or Rhizobiales), Bacteroidota (3 ASVs, comprising one member each of *Cyclobacteriaceae*, *Flavobacteriaceae* and *Rhodothermaceae*), SAR324 clade (2 ASVs), Crenarchaeota (1 ASV, *Candidatus Nitrosopumilus*), Actinobacteriota (1 ASV) and Dadabacteria (1 ASV). All five abundant ASVs associated to nodule‐surface mud were members of γ‐proteobacteria, including *Oceanospirillaceae* and *Alteromonadaceae*.

Many of these abundant ASVs associated to nodules (Figure 4A) or nodule‐surface mud (Figure 4B) were also dominant in the surrounding habitats. However, several abundant ASVs in the nodules or nodule‐surface muds were not or rarely detected in any other habitat type. Hereafter, these ASVs are referred to as nodule‐specific ASVs. As examples of nodule‐specific ASVs, ASV_222 and ASV_305, both of which are members of the SAR324 clade, dominated in the nodule‐surface mud samples (3%–31% at maximum); however, they were rare in other habitat types (up to 0.2%). Similarly, ASV_413 (JTB23), ASV_369 (unclassified γ‐proteobacteria) and ASV_217 (unclassified *Flavobacteriaceae*) were detected exclusively in nodule samples. In contrast, ASV22, ASV52 and ASV196 belonging to Oceanospirales and ASV2 and ASV29 belonging to Alteromonadales were the most abundant in the nodule‐surface mud but were also detected in many nodule samples and few of the other habitat types. Among nodule‐specific ASVs, those belonging to Woeseia were the most notable. Woeseia ASVs, that is, ASV163, ASV_351 and ASV_445, were dominant in many nodule samples, but none were detected in the other habitat types. Interestingly, the other ASVs of Woeseia exhibited distribution patterns different from the above three Woeseia ASVs. For example, ASV19 was detected not only in all nodule and nodule‐surface mud samples but also in many sediment and bottom water samples.

**FIGURE 5:**
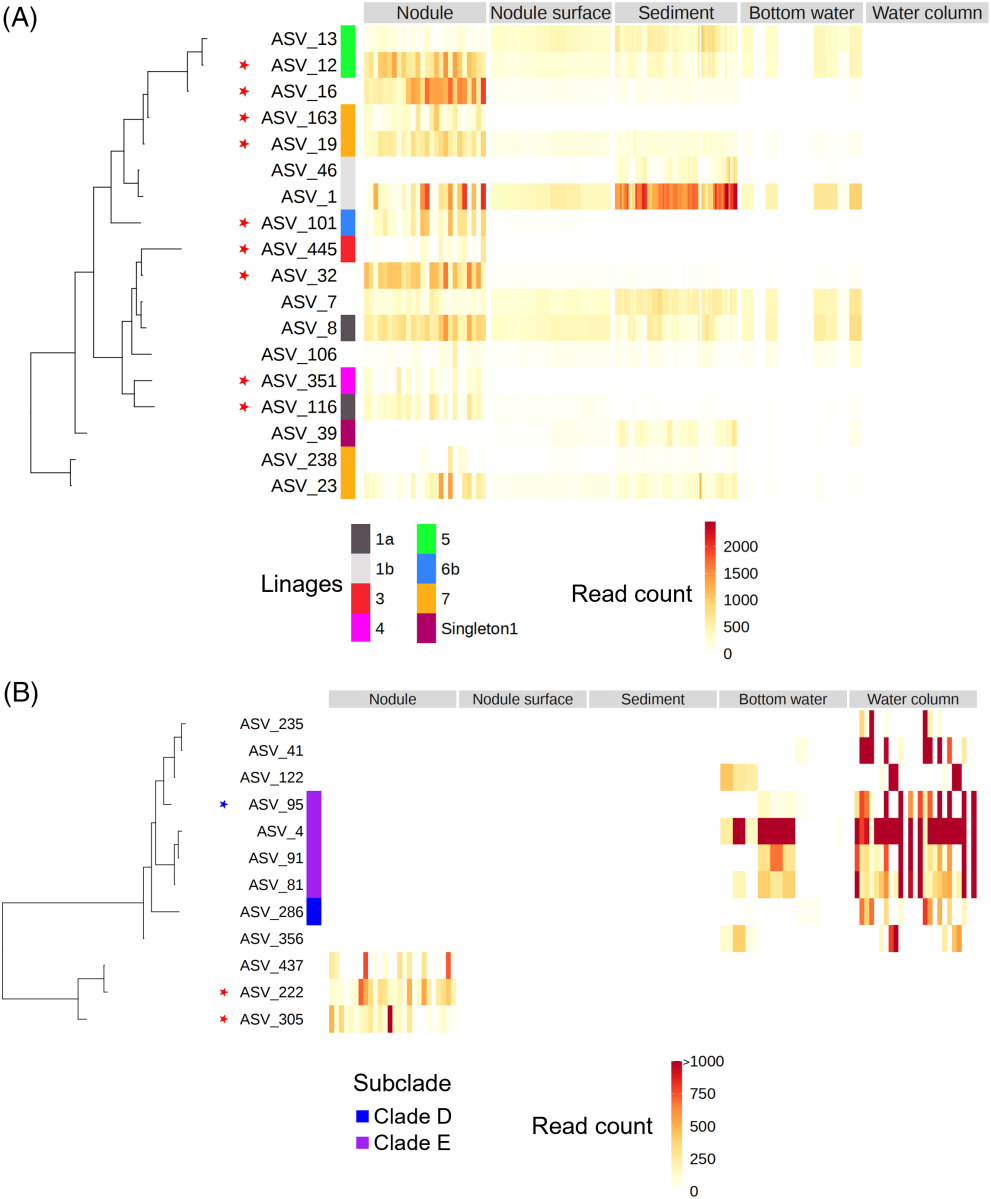
Distribution and phylogeny of nodule‐specific abundant amplicon sequence variants (ASVs) with their relatives. Phylogenetic trees of ASVs were constructed based on V3‐V4 region of 16 rRNA gene sequences. Alignments were performed using MAFFT and trees were inferred using FastTree. The red stars indicate ASVs associated with the specific habitats identified by LefSe. (A) ASVs classified into Woeseiales. Other abundant ASVs classified into gammaproteobacteria were used as outgroups. Linages within Woeseiales were classified based on the phylogenetic placement to a previous study's 16S rRNA gene tree (Hoffmann et al., 2020). (B) ASVs classified into SAR324 clade. ASV classified into NB1‐j were used as outgroups. Subclades within SAR324 were classified based on the phylogenetic placement to a previous study's 16S rRNA gene tree (Boeuf et al., 2021).

To examine whether the nodule‐specific ASVs were phylogenetically distinct from or closely related to the ASVs present in the various habitat types in *Woeseia* and SAR324, we performed phylogenetic placement analyses based on a recent meta‐genome‐based classification of these groups (Boeuf et al., 2021; Hoffmann et al., 2020). Subsequently, 13 of the 18 abundant ASV of *Woeseiales* observed in our study were classified into 7 lineages (1a, 1b, 3, 4, 5, 6b and 7; Figure 5A) based on the classification for *Woeseiales* in a previous study (Hoffmann et al., 2020). These seven lineages have been reported to be associated with deep‐sea environments, except lineage 3, which is more abundant in shallow coastal sediments (Hoffmann et al., 2020). The remaining five ASVs were unclassified or classified into singleton groups, as defined in the previous study. Consistent with these previous reports, all abundant *Woeseiales* ASVs were dominant in samples from the deep sea and sediment, but rare in the water column (Figure 5A). The nodule‐specific ASVs of *Woeseia*, ASV163, ASV_351 and ASV_445 were classified as lineages 7, 4 and 3, respectively. Although ASV445 and ASV 351 were the sole representative ASVs of lineages 3 and 4, respectively, in this study, lineage 7 also included three ASVs, namely ASV_19, ASV_23 and ASV238, showing broader distributions, including sediment samples (Figure 5A).

In SAR324, except for nodule‐specific ASVs, abundant ASVs were rare in sediment or nodules, but were present in water samples (Figure 5B). Phylogenetic placement analysis classified only 4 of the 18 abundant ASVs into previously described subgroups (D or E, Boeuf et al., 2021), and nodule‐specific ASVs were not classified into any known subgroups. While the phylogenetic tree constructed from the V3‐V4 region of 16S rRNA gene had limited information for accurate classification, the nodule‐specific ASVs were distinct from other abundant ASVs that dominated in water samples in the phylogenetic tree (Figure 5B).

## DISCUSSION

Recently, prokaryotic activities have been suggested to contribute to nodule formation as one of the core mechanisms of nodule formation; however, the relationship between the nodule/crust formation process and prokaryotic activity remains unclear (Blöthe et al., 2015; Kato et al., 2019; Wang & Müller, 2009; Wu et al., 2013). In this study, we identified populations that were highly and specifically enriched in nodule habitats at the intraspecies‐level via high‐throughput sequencing of 16S rRNA gene under ASV‐level resolution. Interestingly, taxa of these nodule‐specific ASVs, such as members of SAR324 and *Woeseia* found in this study, have also been reported to dominate marine benthic environments but are not specific to nodule fields in previous studies (Boeuf et al., 2021; Hoffmann et al., 2020). Our observations demonstrated that specific populations of these taxa were highly enriched in the nodule, but not in any other investigated habitat. In addition, we identified several ASVs that were enriched in muds attached to the nodule surfaces. The nodule‐surface mud‐associated ASVs include *Alteromonas*, of which some strains are able to oxidize Mn (Templeton et al., 2005) and thus could play a role in inducing or controlling the mineralization of Mn (Wu et al., 2013). Thus, the particular populations of these taxa were highly likely to be associated with nodules. Similar small‐scale specialization of particular populations in marine sedimentary mineral deposits was also reported in the Fe‐Mn crust, and such specialized populations in marine deep‐sea sediments would thrive by utilizing available particulate organic matter sunk from the water column (Kato et al., 2018). These ASVs, which are associated with nodules or nodule‐attaching muds, are candidates for taxa involved in nodule formation and are promising targets for future studies to examine microbial processes in nodule fields.

Similar studies have been performed in the CCZ (Lindh et al., 2017) and other areas that detected nodule‐related prokaryotes (Bergo et al., 2021; Kato et al., 2018; Liu et al., 2019). However, those studies investigated prokaryotic community structures at the nodules and surrounding environments in the OTU‐level corresponding to “species.” Compared with previous observations of prokaryotic communities, members of the dominant taxa detected as nodule‐specific ASVs in our study, such as SAR324, were commonly detected in other studies (Bergo et al., 2021; Kato et al., 2018; Liu et al., 2019). Although the previous studies suggests SAR324 was the “generalist” lineage of which OTUs were detected in both nodules and the surrounding environment (Lindh et al., 2017), our ASV‐resolution analysis revealed the existence of nodule‐specific SAR324 populations which are distinct from the known deep layer clade of SAR324 (Figure 5). Our ASV‐level resolution analysis suggests the existence of niche specialization in SAR324, and few are highly specialized in nodule environments. Another notable difference compared with previous study in the CCZ, Cyanobacteria, which were the most abundant taxa in the epipelagic zone and often detected in nodules in a previous study (Lindh et al., 2017), were relatively less abundant in the epipelagic zone and very rare in nodules at our sampling stations the 3 years observation. This is presumably due to the occurrence of seasonal picocyanobacterial blooms in the previous study and they seem to be not endemic taxa of nodules.

A previous study reported an increase in bacterial abundance after 1 year of the JET disturbance experiment (Fukushima, 1995), and a recent assessment of the effect of the disturbance experiment carried out in 1989 in the Peru Basin nodule field suggests the existence of a remaining impact on the microbial activity after 26 years (Vonnahme et al., 2020). However, in our observations, no statistically significant differences in alpha and beta diversity were observed between the samples obtained from the heavy deposition area (J6‐08) and other stations among the same habitat type samples. This may suggest that the effects of the disturbance experiment of JET experiment on microbial community diversity no longer be detectable within 30 years after the experiment. However, because our study did not monitor biomass and microbial activity, a comprehensive assessment of these factors is still needed to accurately assess the impact of deep‐sea mining on the prokaryotic community.

## CONCLUSION

This study highlights the importance of discriminating prokaryotic taxa in ASV‐level resolution to reveal genuine Fe‐Mn nodule‐associated prokaryotic populations. These nodule‐specific populations are promising targets for future studies to examine the microbial contribution to the nodule formation process.

## AUTHOR CONTRIBUTIONS

**Kento Tominaga:** Formal analysis (lead); methodology (equal); writing – original draft (lead). **Hiroaki Takebe:** Formal analysis (supporting); methodology (supporting); writing – review and editing (equal). **Chisato Murakami:** Conceptualization (equal); project administration (equal); supervision (equal); writing – review and editing (equal). **Akira Tsune:** Project administration (equal); supervision (equal); writing – review and editing (equal). **Takahiko Okamura:** Data curation (equal); formal analysis (supporting); methodology (equal); visualization (supporting); writing – review and editing (equal). **Takuji Ikegami:** Project administration (equal); resources (equal); writing – review and editing (equal). **Yosuke Onishi:** Project administration (equal); resources (equal); writing – review and editing (equal). **Ryoma Kamikawa:** Project administration (equal); writing – review and editing (lead). **Takashi Yoshida:** Conceptualization (equal); funding acquisition (equal); project administration (equal); resources (equal); supervision (lead); writing – review and editing (lead).

## CONFLICT OF INTEREST STATEMENT

The authors declare no conflicts of interest.

## Supporting information


**Data S1.** Supporting Figures.

**Table S1.** Metadata and statistics of samples analysed in this study.

**Table S2.** Feature count table of amplicon sequence variants analysed in this study.

**Table S3.** Detailed statistics of alpha and beta diversity analysis. Statistically significant results are highlighted in red.

## Data Availability

Sequences obtained from the observations were deposited in the DNA Data Bank of Japan (DDBJ) under project number PRJDB14745. Raw sequence reads can be found under accession numbers SAMD00555457 to SAMD00555598.
