# Supplementary material for: Population‐level prokaryotic community structures associated with ferromanganese nodules in the Clarion‐Clipperton Zone (Pacific Ocean) revealed by 16S rRNA gene amplicon sequencing
Source: Environ Microbiol Rep. 2023 Dec 26;16(1):e13224. doi: 10.1111/1758-2229.13224 (PMC10866075; doi:10.1111/1758-2229.13224)
Supplement: Supplementary file 1 — Data S1. Supporting Figures. [file EMI4-16-e13224-s002.pdf]

Supporting Information

Supporting Figures

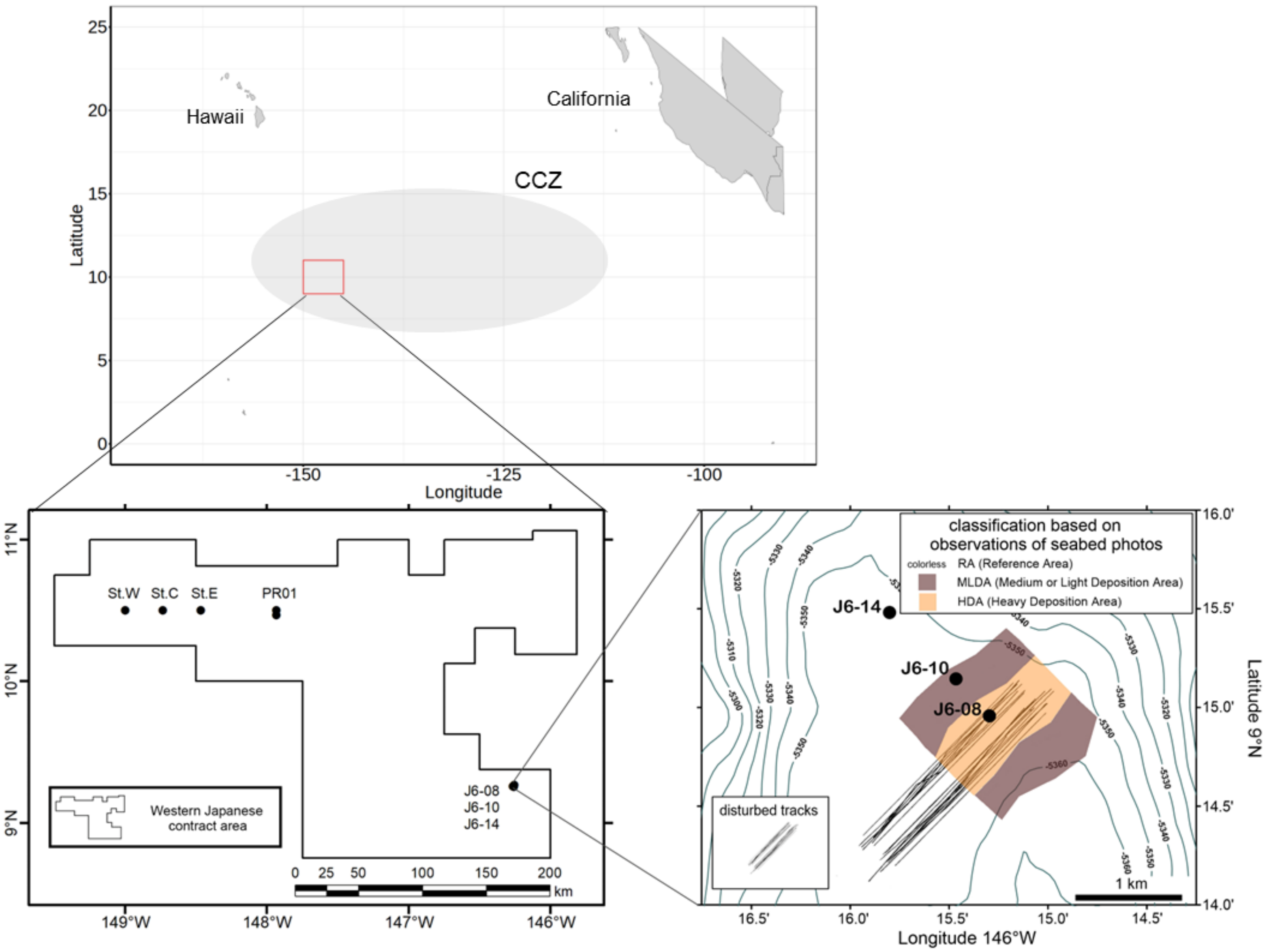

Figure S1 Map of the sampling site in this study.

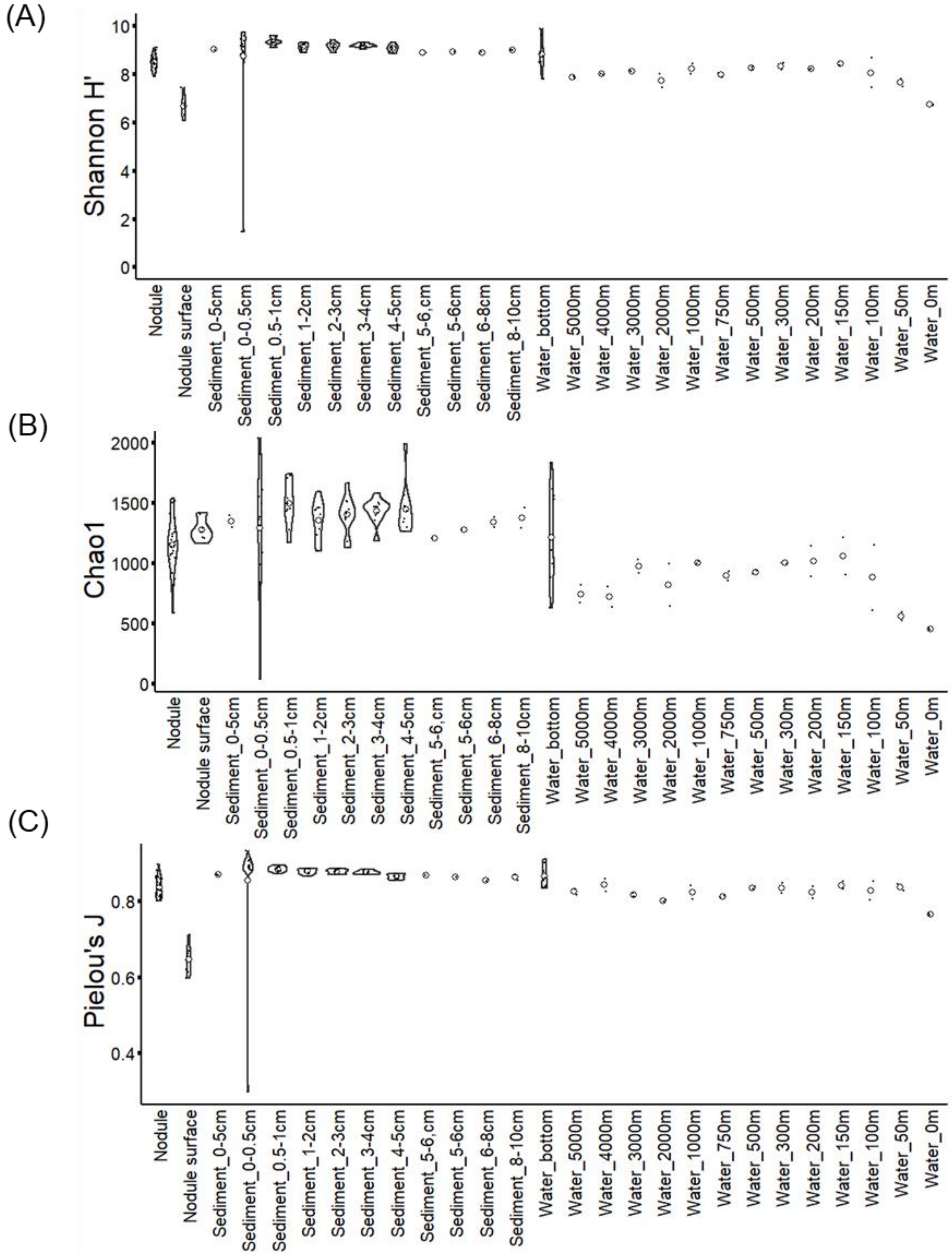

Figure S2 Alpha diversity indices in detailed sample types (sediment layers and depth). (A) Shannon H' (Richness and evenness). (B) Chao1 (richness). (C) Pierou's J (evenness).

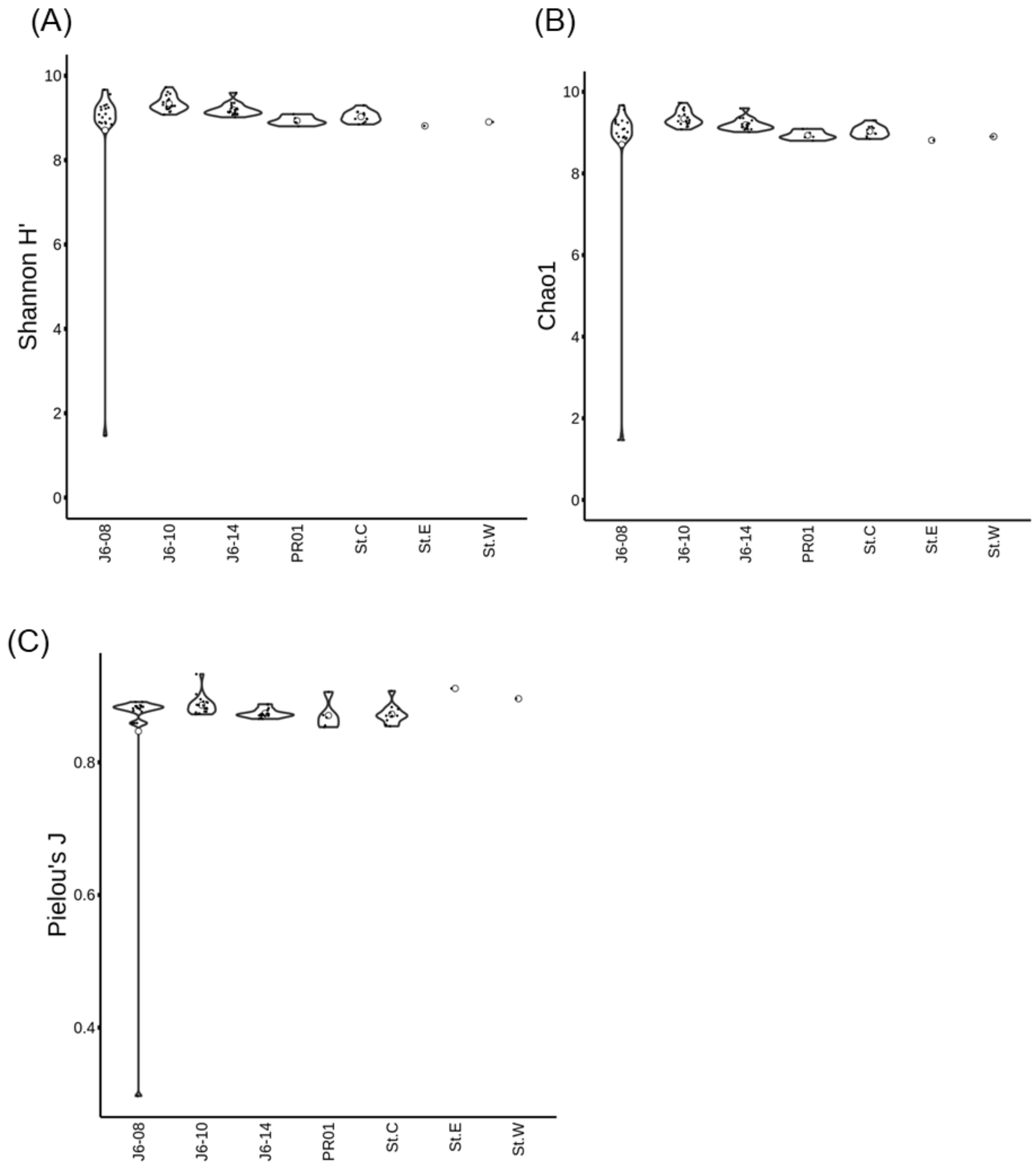

Figure S3 Comparison of alpha diversity indices between different stations in sediment samples. (A)

Shannon H' (Richness and evenness). (B) Chao1 (richness). (C) Pierou's J (evenness).

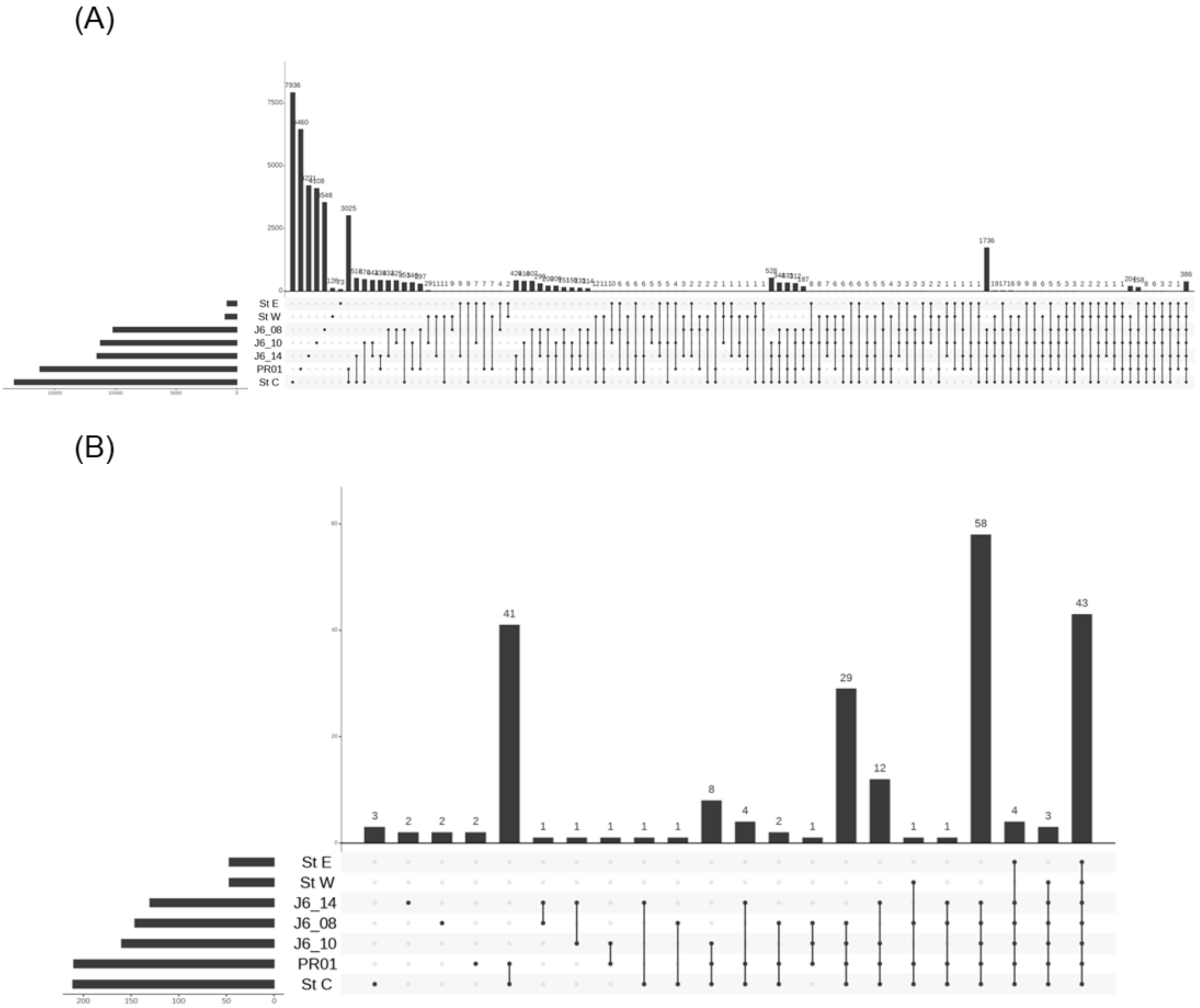

Figure S4. Upset plot showing the distribution of amplicon sequence variants (ASVs) and the number of shared ASVs between sampling station. (A) All ASVs. (B) Only abundant ASVs (exceeding >1% relative abundance at least a sample).

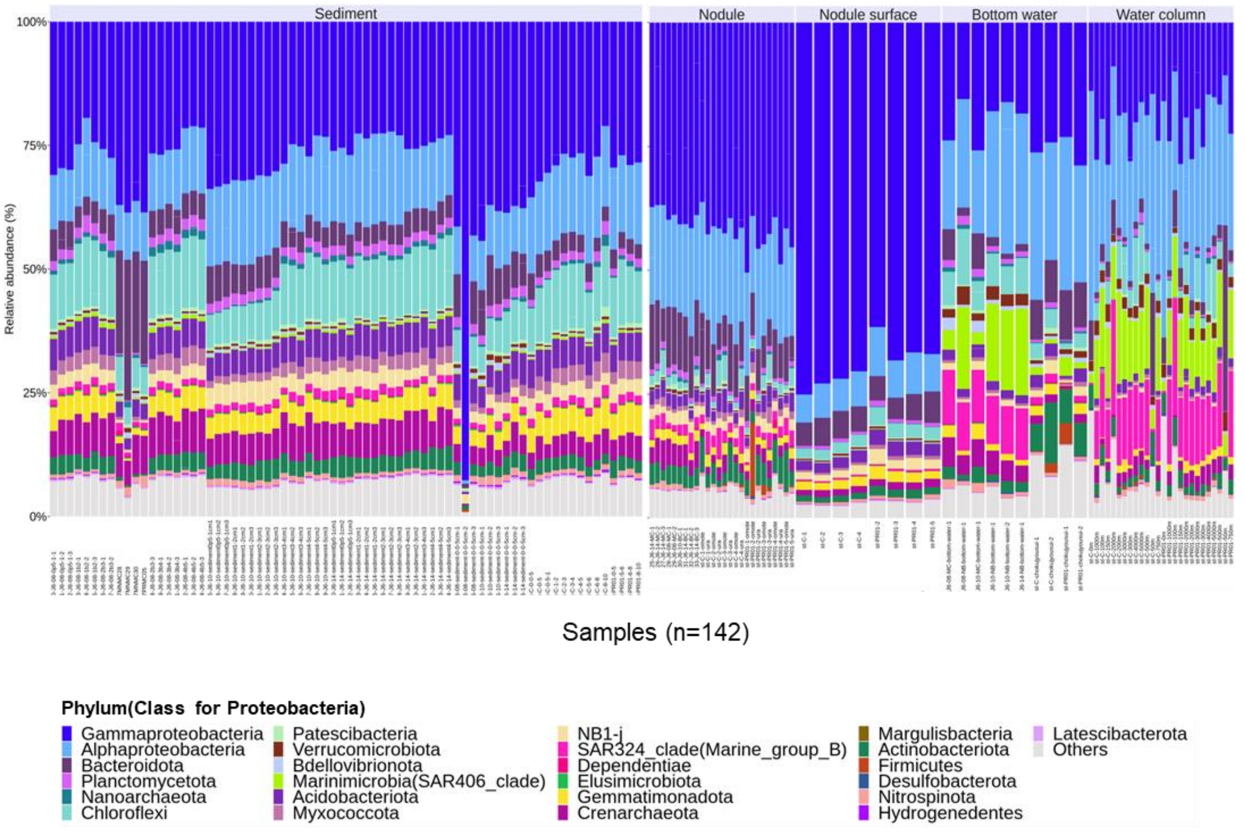

Figure S5 The relative abundance of prokaryotic taxonomic composition for phylum or class (for

proteobacteria) level in 142 samples.

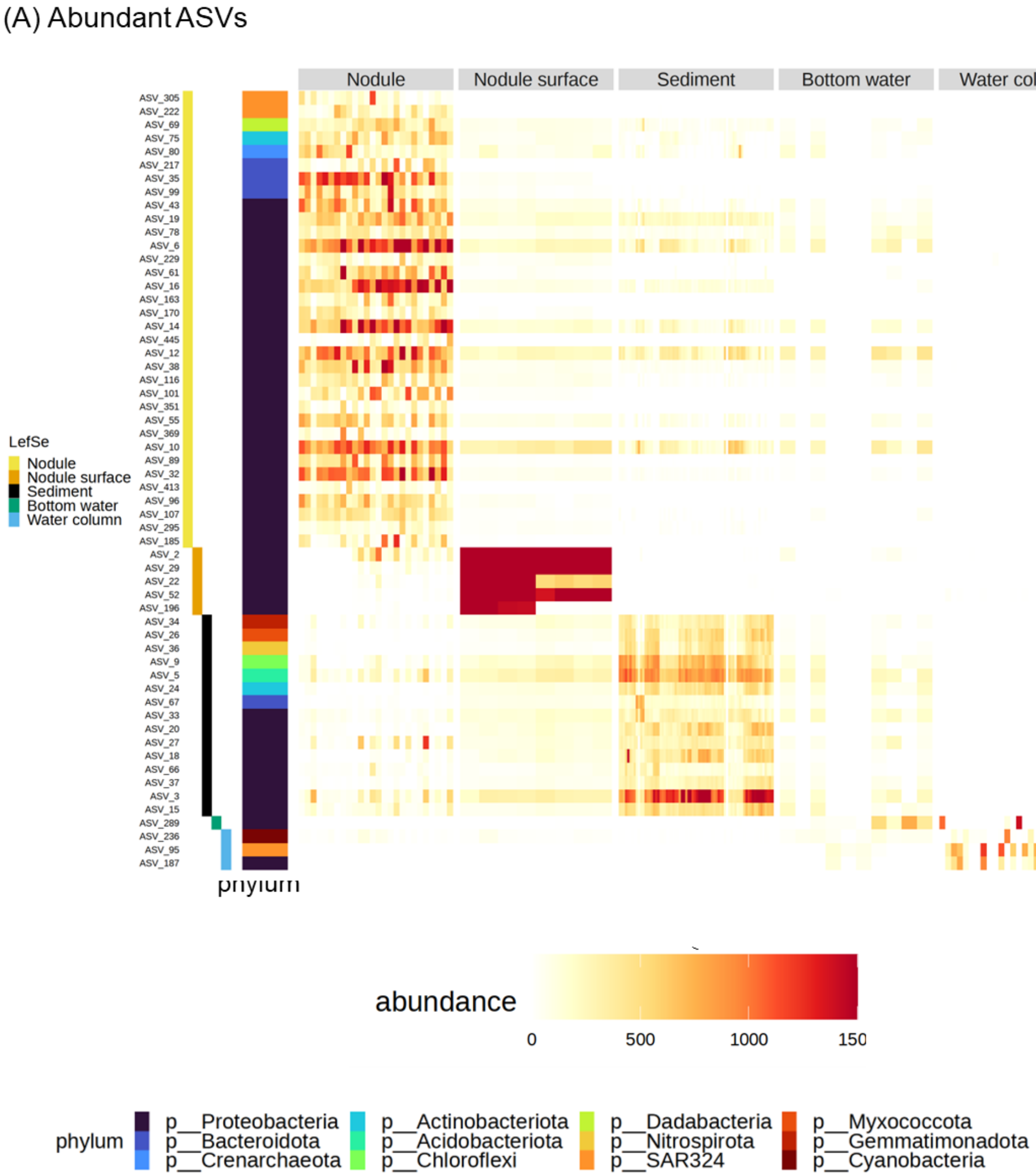

(B) Others

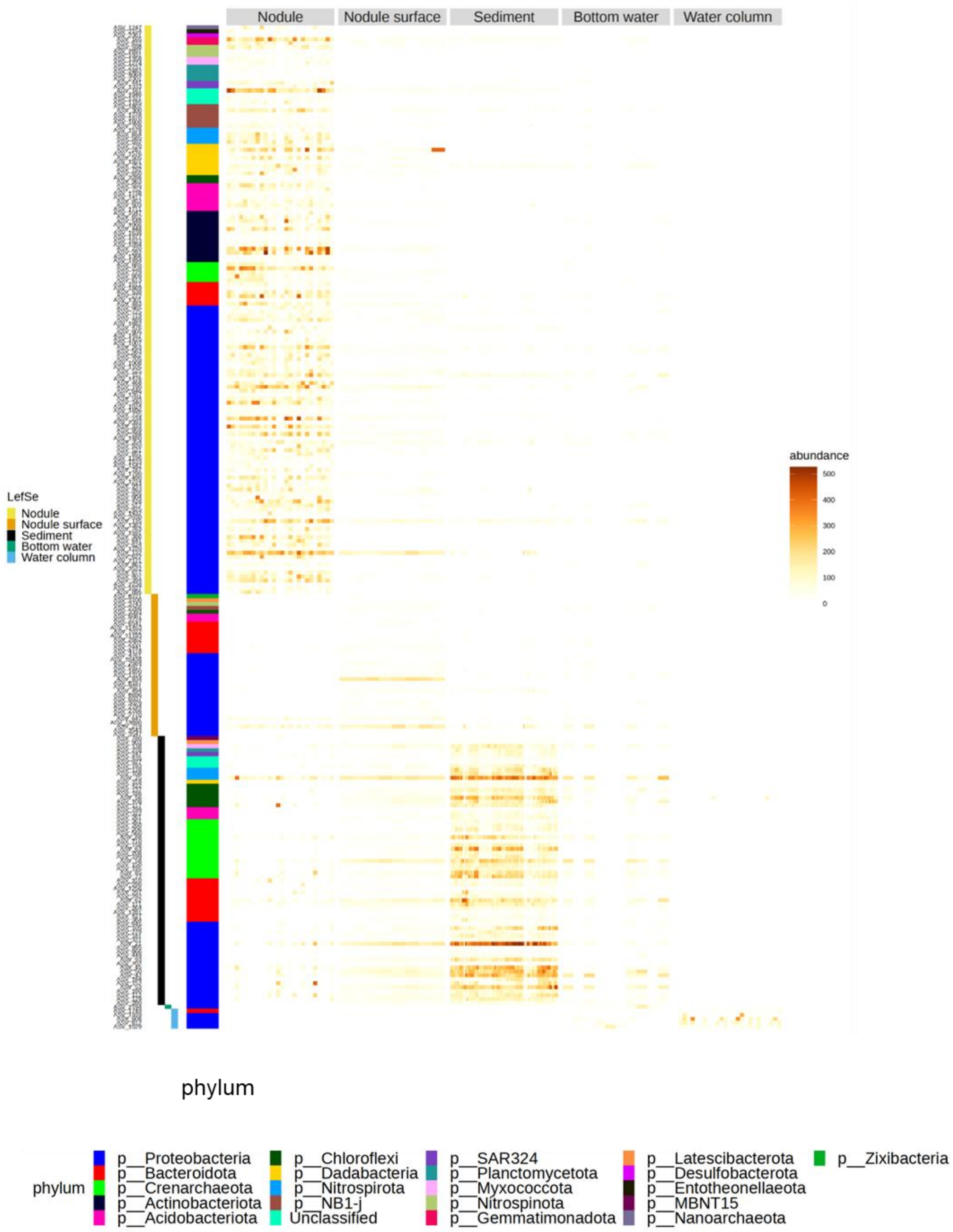

Nodule
Nodule surface
Sediment
Bottom water
Water column
LefSe
Nodule
Nodule surface
Sediment
Bottom water
Water column
abundance
500
400
300
200
100
0
phylum
phylum
p__Proteobacteria
p__Bacteroidota
p__Crenarchaeota
p__Actinobacteriota
p__Acidobacteriota
p__Chloroflexi
p__Dadabacteria
p__Nitrospirota
p__NB1-j
Unclassified
p__SAR324
p__Planctomycetota
p__Myxococcota
p__Nitrospinota
p__Gemmatimonadota
p__Latescibacterota
p__Desulfobacterota
p__Entotheonellaeota
p__MBNT15
p__Nanoarchaeota
p__Zixibacteria

Figure S6 Abundance of sample-associated amplicon sequence variants (ASVs) selected by LefSe.

The heatmap divided by sample types represents the abundance of each ASV in the samples. Color bars next to the ASV names represent associated sample types determined by Lefse. A Color bar next to the heatmap represents the phylum of each ASV. (A) Abundant ASVs (B) Other ASVs.

Supporting Tables

Table S1 Metadata and statistics of samples analyzed in this study.

Table S2 Feature count table of amplicon sequence variants analyzed in this study.

Table S3 Detailed statistics of alpha and beta diversity analysis. Statistically significant results are highlighted in red.
